# Acquiring the Diagnosis of an Acquired Tracheoesophageal Fistula with EtCO_2_: A Case Discussion with Review of the Literature

**DOI:** 10.1155/2023/9437558

**Published:** 2023-03-20

**Authors:** Janine Beatrice Borja, Ghanshyam Patel, Luqman Baloch, Ammar Aqeel, Mool Chand, Hariprasad Korsapati, Altaf Dawood, Naser Khan

**Affiliations:** ^1^Mercyhealth Internal Medicine Residency, Javon Bea Hospital, Rockford, IL, USA; ^2^Mayo Clinic Health System, Mankato, MN, USA; ^3^Hospitalist Services, Javon Bea Hospital, Rockford, IL, USA; ^4^Medical Director of Mercyhealth Gastroenterology Services, Javon Bea Hospital, Rockford, IL, USA

## Abstract

Acquired tracheoesophageal fistula (TEF) is a rare complication of esophageal or lung cancer. A 57-year-old male presented with complaints of vomiting, cough, 20 lb weight loss, and progressive dysphagia. Early laryngoscopy and CT chest showed a normal pharynx with an irregular thickness of the thoracic esophagus. The upper gastrointestinal endoscopy (UGIE) and upper endoscopic ultrasound (EUS) revealed a hypoechoic mass evolving as complete obstruction. During the procedure, minimal CO_2_ was used for insufflation; however, when attempts were made to traverse the obstruction, capnography revealed an end-tidal CO_2_ (EtCO_2_) estimating 90 mmHg indicating possible TEF. This case depicts the use of capnography during UGIE in diagnosing an acquired TEF.

## 1. Introduction

TEF is the abnormal connection between the esophagus and the trachea. Emergent diagnosis and treatment are required as gastric contents contaminate bronchial trees leading to respiratory failure, sepsis, and death. TEFs can be congenital or acquired. Congenital causes are rare and accompanied by tracheal atresia or stenosis. Acquired TEF causes include iatrogenic injury, prolonged mechanical ventilation, malignancy, tracheostomy, trauma, foreign body, and mediastinitis.

In a patient with upper gastrointestinal (GI) malignancy, the TEF may not present with typical signs and symptoms. An extreme index of suspicion is therefore needed to identify TEF. The diagnostic modalities include barium swallow, CT imaging, bronchoscopy, and UGIE. A contrast esophagogram is the preferred diagnostic modality. CT is also helpful to characterize the fistula and airway anatomy. However, CT may not be helpful to diagnose the extent and precise location of TEF. Further evaluation with upper GI endoscopy will help find the exact location and extension of TEF. Treatment options are surgical and nonsurgical, depending on the location and severity of TEF. The life expectancy of TEF patients without early surgical repair may be measured in weeks.

## 2. Case Description

A 57-year-old male with a medical history of hypertension, alcohol misuse, and ongoing tobacco use (40 pack-years) presented to primary care with complaints of dysphagia 2 years ago. The patient had a sensation of a lump in his throat but denied any reflux symptoms. He also complained about difficulty in swallowing solids. Vital signs, physical examination, and blood work at the time did not reveal any alarming concerns. He was suspected of having oropharyngeal dysphagia. Laryngoscopy revealed normal pharynx, erythema in the arytenoid, and esophageal inlet without any mass. Thereafter, the patient was lost to follow-up due to the COVID-19 pandemic.

Several months later, he presented to the emergency department with complaints of vomiting, productive cough, and fatigue. The patient was looking cachectic and had unintentional weight loss of 20 lb. The symptom of dysphagia gradually progressed to liquid over 2 years. At this point, the patient was suspected to have esophageal cancer. Initial CT chest showed irregular wall thickening involving 5 cm length of mid-esophagus, enlarged subcarinal lymph nodes, and a 1.5 cm pulmonary nodule in the right middle lobe.

**Figure 1:**
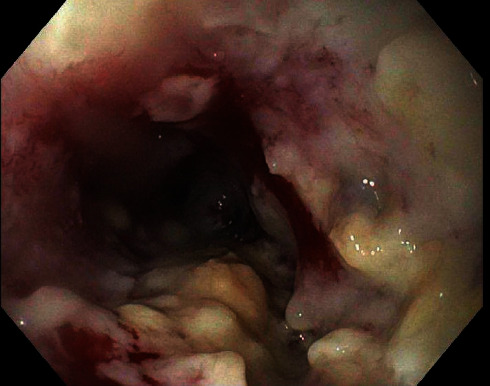
Large ulcerating mass in the middle third of the esophagus, 26 cm from the incisors on the UGIE.

Due to suspicion of esophageal cancer, he underwent UGIE. The patient was found to have a large ulcerating mass with stigmata of recent bleeding on the middle third of the esophagus, 26 cm from the incisors (Figure 1). The mass was completely obstructing and partially circumferential involving two-thirds of the lumen circumference. Biopsies were taken with cold forceps for histopathology. The endoscope could not traverse due to near-complete obstruction. A limited upper endoscopic ultrasound (EUS) evaluation was performed from the proximal edge of the lesion revealing a hypoechoic mass with irregular borders in the middle third of the esophagus, involving 100% of the lumen circumference, measuring up to 15 mm in thickness. An evaluation was limited due to obstruction. The proximal portion of the esophageal mass was noted to be invading adventitia, making it at least T3 lesion. Histopathology revealed invasive poorly differentiated squamous cell carcinoma.

**Figure 2:**
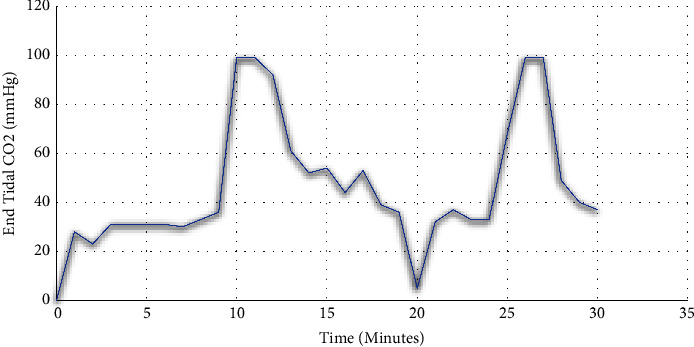
Capnography showing an abrupt end-tidal CO_2_ rise to 99 mmHg with full insufflation of the esophagus with carbon dioxide (CO_2_), suggesting the presence of TEF.

During the procedure, minimal CO_2_ was used for insufflation, but when attempts were made to traverse the obstruction, capnography revealed an end-tidal CO_2_ (EtCO_2_) estimating 90 mmHg indicating a high likelihood of TEF (Figure 2 showing the pattern of end-tidal CO_2_ on capnography during endoscopy). A fluoroscopic GI esophagram with barium swallow was carried out to confirm the diagnosis of TEF. The patient took one sip of thin barium and immediately had an episode of emesis with a significant amount of contrast noted within the bronchial tree, so the exam was terminated. A proximal esophageal stent was placed with upper endoscopy under fluoroscopic guidance. Also, a percutaneous endoscopic gastrostomy (PEG) tube was placed for tube feeding. Nutrition education was provided for home tube feeding. The patient was discharged home with intermittent skilled nursing care, physical therapy, and speech therapy.

## 3. Discussion

Over 50% of acquired TEFs or mediastinal fistulas are a complication of esophageal cancer [1]. Esophageal cancer is the seventh most common cause of cancer death among men accounting for 1% of cancers diagnosed in the United States [2]. Usually, TEF is not suspected when esophageal cancer is in the differential diagnosis. We report a case of TEF secondary to the middle third of esophageal squamous cell carcinoma, which was diagnosed unconventionally. The fistula was not visualized on CT imaging; also during UGIE with EUS, the view was obscured due to complete esophageal lumen obstruction. On esophagoscopy, the hypoechoic 15 mm circumference mass was observed, which was occupying 100% of the lumen circumference. When attempts were made to pass the endoscope through the obstruction, capnography showed a sudden rise in EtCO_2_ from 40 mmHg to 90 mmHg. This unique finding eventually led to the diagnosis of TEF unconventionally.

Diagnosing TEF is difficult in patients with esophageal mass and requires a high index of intuition. Capnography is a tool for measuring metabolic and respiratory functions, which monitors the CO_2_ concentration in the atmosphere. This is the standard technique used while endoscopy, which provides breath-by-breath analysis and records continuous ventilatory status while procedures. The spike in EtCO_2_ while esophagoscopy uses CO_2_ insufflation facilitated the identification of TEF due to gas passage from the esophagus to the trachea. Identification of TEF can be challenging, as many a time, esophagoscopy may miss small or subtle fistulas even with aided fluoroscopy. The esophagram with barium is the confirmatory test for TEF. Though symptoms of TEF are vague, physicians need to be cognizant in diagnosing TEF as it can lead to respiratory failure. Most of the time, TEF in patients with GI malignancy present late; thus, the treatment usually focuses on palliative care. In those circumstances, the treatment depends on the surgical resectability of the primary tumor and trajectory of the fistula [3]. Iatrogenic or benign TEFs receive conservative treatment; however, depending on location and dimensions, it may necessitate surgical reconstruction. In our case, the diagnosis of TEF helped us to have change in approach for the treatment with proximal stent placement, which may improve survival.

EtCO_2_ is a standard inexpensive monitoring tool that helps us to find out hypoventilation, hypermetabolic states, and mechanical ventilator malfunction. Throughout the continuous monitoring of EtCO_2_ in the capnogram, any aberrant fluctuations denote underlying etiology. The excessive increment in EtCO_2_ with rapid return to the baseline after CO_2_ insufflation during esophagoscopy confirms the TEF as the underlying etiology. The identification of TEF might have been delayed if this finding on the capnogram was not visualized as the 15 mm circumferential mass in the middle third esophagus was obscuring the view on esophagoscopy. This case accentuates the significance of capnography as a diagnostic tool for nonevident fistulas between the trachea and esophagus.

The standard treatment options for acquired TEF may include airway stent, fibrin glue injections, and over-the-scope clipping. A retrospective study of 123 patients on survival and outcome of esophagorespiratory fistulas (ERF) comparing the surgical intervention to nonsurgical treatment concluded that survival in malignant ERF is better with the surgical intervention [4]. Lenz et al. also described that patient with malignant ERF who underwent nonsurgical treatment had an increased risk of death compared with those who underwent surgery exploring a hazard ratio of 5.5 [4].

Detecting openings between the esophagus and trachea remains challenging. The combination of EtCO_2_ during endoscopy with CO_2_ insufflation is a unique and cost-effective way to improve diagnostic modality for TEF. Yasuda et al. hypothesized combination of capnography ≥68 mmHg with esophagoscopy, fluoroscopy, and bronchoscopy compared to esophagoscopy alone or bronchoscopy alone has the highest diagnostic sensitivity for TEF recognition (sensitivity 88.1%) [5]. More research studies are required on diagnostic modalities for TEF as standard diagnostic algorithms have not been well described. We hypothesize the retrospective study could empower more precise differentiation by using the rapid increase in EtCO_2_ during esophagoscopy for TEF identification.

## Data Availability

No data were used to support this study.
